# Effects of a Long-Term Exercise Training Program on the Functional Capacity and Health-Related Quality of Life in Inpatients with Psychotic Disorders: A Randomized Controlled Trial

**DOI:** 10.3390/jfmk10040401

**Published:** 2025-10-15

**Authors:** Victoria Theochari, Dimitra Mameletzi, Andriana Teloudi, Stergios Kaprinis, Evangelia Kouidi

**Affiliations:** 1 Laboratory of Sports Medicine, Department of Physical Education and Sports Science, Aristotle University of Thessaloniki, 57001 Thessaloniki, Greece; victoriatheohari@gmail.com (V.T.); mamel@phed.auth.gr (D.M.); teloudia@phed.auth.gr (A.T.); 2 Laboratory of Psychophysiology, 3rd Department of Psychiatry, Aristotle University of Thessaloniki, University Hospital AHEPA, 54124 Thessaloniki, Greece; skaprinis@auth.gr

**Keywords:** exercise, functional ability, quality of life, psychotic disorders, Pilates

## Abstract

**Background**: This randomized controlled trial aimed to assess the effects of a yearly Multi-Component Exercise Training (MCET) program performed within the hospital setting on the functional capacity and Health-Related Quality of Life (HRQoL) of inpatients with psychotic disorders. **Methods**: Forty-eight inpatients of a public Psychiatric Hospital with a diagnosis of psychosis participated in the study and were randomly assigned to two groups: (i) the intervention group (mean age: 46.6 ± 13.2) and (ii) the control group (mean age: 47.7 ± 8.9). The MCET program consisted of moderate-intensity aerobic exercise (AE), Pilates exercises, and strengthening, balance, corrective and flexibility exercises, using rubber bands, mobility sticks and balls. Sessions were implemented three times per week over a 12-month period and each group session lasted for 30–45 min. The primary outcomes of the study included functional capacity and HRQoL. Functional capacity was assessed through measurements of strength (using handgrip and leg dynamometer), balance, and body positioning, while HRQoL was evaluated using the 36-Item Short Form Health Survey (SF-36). **Results**: At the end of the treatment, participants in the intervention group demonstrated significantly increased lower and upper extremity muscle strength by 35.9% (*p* < 0.01) and 23.6% (*p* < 0.01), respectively, improved balance by 18.1% (*p* < 0.001), and enhanced sit-and-reach and sit-to-stand results by 47.6% (*p* < 0.001) and 18.2% (*p* < 0.001), respectively, as well as increased distance covered at 6MWT by 26.2% (*p* < 0.001). Regarding the HRQoL, all the parameters of the SF-36 were improved, including general and mental health (*p* < 0.05), physical (*p* < 0.001) and social function (*p* < 0.05), physical (*p* < 0.01) and emotional roles (*p* < 0.01) and vitality and bodily pain (*p* < 0.001 and *p* < 0.05, respectively). The Physical Component Summary score (PCS) was enhanced significantly (*p* < 0.001), while the Mental Component Summary score (MCS) remained unchanged. Compliance with the 1-year ET program was 80%. **Conclusions**: The findings of the study indicated that a 1-year moderate-intensity MCET performed three times per week was safe, well accepted and efficient in improving physical functioning and HRQoL among inpatients with psychotic disorders. These results suggest that structured exercise interventions could be prescribed as add-ons to the pharmacological treatment for psychotic disorders.

## 1. Introduction

Mental health is an important component of overall health, multiplying mortality risk [1], affecting health-related quality of life (HRQoL) [2] and disability [3] of patients, while increasing healthcare costs [4]. According to the World Health Organization [5], mental health consists of a universal public health priority alongside non-communicable diseases. In alignment with this perspective, the European Union [6] aims to promote mental well-being and prevent mental health disorders, thereby recognizing mental health as equally important as physical health.

The growing concern for mental wellbeing has increased interest in the implementation of exercise training (ET) protocols for people with mental health issues. Today, a plethora of scientific evidence highlights the harmful effects of physical inactivity in health, indicating that exercise is, in fact, medicine [7]. Appropriate ET programs can improve overall health, including cardiovascular risk, as well as psychological wellbeing [8]. Participation in ET has been shown to reduce depression [9], ameliorate physical self-worth, mental health and wellbeing, resilience, self-esteem and self-efficacy [10]. In parallel, it has been shown to improve HRQoL in schizophrenia spectrum and several other psychotic disorders [11]. For individuals with psychotic disorders, participation in moderate-intensity ET, performed several times per week, has been suggested to induce positive effects [12]. Recently, Ziebart [13] indicated that aerobic exercise (AE) implemented in the hospital setting is efficient in reducing negative and symptom severity scores in psychotic disorders. Despite these promising findings, a scientific gap remains in the literature regarding the longitudinal effects of ET in patients with psychotic disorders. According to the literature, previous studies reported intervention durations ranged from 4 to 24 weeks, with an average of approximately 12 weeks, providing insufficient data on the sustainability of a study over a more extended period [13,14,15]. Furthermore, a limited number of studies have examined exercise programs within the hospital settings, where patients often experience prolonged institutionalization, sedentary lifestyle and long-term exposure to antipsychotic medication. Therefore, the originality of the present study mainly lies in the extended duration of the protocol, which exceeds that of most previous interventions and in the fact that long-term ET interventions within hospital settings remain unexplored. This Randomized Controlled Trial (RCT) aimed to assess the effects of a long-term MCET program performed inside the hospital setting, on the functional ability and Health-Related Quality of Life (HRQoL) in inpatients with psychotic disorders. Based on the existing literature, we hypothesized that participation in the 12-month MCET program would lead to significant improvements in functional capacity, and secondarily, that it would also result in significant improvements in HRQoL, assessed be SF-36.

## 2. Materials and Methods

### 2.1. Study Protocol and Ethical Approval

The research question was “what is the effect of participation in a MCET program performed within the hospital setting thrice per week, on the functional ability and HRQoL in patients with psychotic disorders, compared to no ET?”

The study protocol was evaluated and approved by the Ethics Committee of the Department of Physical Education and Sports Science of Aristotle University of Thessaloniki (Protocol number: EH-44/2021). Furthermore, the clinical trial was registered on ClinicalTrials.gov (NCT07113119).

### 2.2. Patients and Eligibility Criteria

The present study was conducted at the Multi-Purpose Nursing Unit of Mental Health, which is based in the city of Thessaloniki in northern Greece and constitutes a decentralized unit of the Regional Network of Mental Health Services (R.N.o.M.H.S.). Inclusion criteria: involved (i) being an adult, (ii) inpatient with a diagnosis of psychotic syndrome, (iii) on stable medication, (iv) controlled as far as psychosis is concerned, and (v) consenting to participate. Exclusion criteria involved: (i) adolescents, (ii) with other diagnoses, (iii) not on stable medication, (iv) in an unstable condition, and (v) unwilling to participate in the study. Following the baseline assessment, a total of 48 eligible inpatients were enrolled in the study. Participants were randomly allocated, in a 1:1 ratio using the online platform www.randomizer.org (accessed on 31 August 2021), to either (i) the intervention group or (ii) the control group. The intervention group (Group A, n = 24) participated in a structured 12-month MCET program, whereas the control group (Group B, n = 24) received usual care. The medication regimen remained constant during the study. Furthermore, approval for participation in the specific MCET program of this study was provided by the attending physician and the patients who were going to participate were informed about the purpose and procedures of the study. Thereafter, they completed the “Consent Form in Research”, which was the formal prerequisite for their participation. All procedures were conducted in accordance with the regulations of the Ethics Committee DPO (Data Protection Officer) of the Multi-Purpose Nursing Unit of Mental Health in Thessaloniki, in compliance with the stipulated rules and instructions. Study participants were blinded to group allocation until the beginning of the ET intervention. All the outcome assessments were conducted by independent evaluators who were blinded to group allocation.

### 2.3. Intervention and Comparison

The 1-year MCET program was implemented in the organized gym of the Multi-Purpose Nursing Unit of Mental Health in Thessaloniki and was conducted by a physical education instructor experienced in exercise-based rehabilitation. The exercise program was tailored to the needs and capabilities of this patient population and was carefully structured, in accordance with current guidelines, to ensure the safety of the participants. Patients in the intervention group participated in a moderate-intensity exercise program which was performed 3 times per week, and they were required to attend at least 80% of all sessions during the total 12-month period. Each session began with a 10-min warm-up period, which included dancing or walking and stretching exercises, and ended with a 5-min relaxation period, which included stretching and breathing exercises. The core program was 30 min of MCET and consisted of 10 min of AE, 10 min of Pilates and 10 min of strengthening, balance, corrective and flexibility exercises, which were performed with rubber bands, mobility sticks and small Pilates balls. The Pilates exercises performed by the patients were of moderate intensity, i.e., at 60–75% maximum heart rate (HR) and the most appropriate ones were selected in order not to cause risks to them, nor injuries.

Progression of the training load was implemented gradually, beginning with an increase in the number of repetitions and subsequently by the addition of sets, in accordance with the participant’s progress and safety considerations. The intensity and frequency of the exercises gradually increased as various adjustments were made to the training. All sessions were delivered to small groups of 6–7 participants, ensuring appropriate guidance, safety and adaptation of the exercise to the collective needs and abilities of the group. Nevertheless, the intensity and the type of exercise could be adjusted according to each participant’s personal progression and limitations. During the training sessions, heart rate and blood pressure were measured at each break between activities. The patients also assessed their perceived effort on the Borg scale and were constantly encouraged to reach their perceived effort at level 13–14 of the Borg 6–20 category scale (which concerns the assessment of the subjective intensity of the exercise during its performance and ranges from 6—no effort, to 20—maximum effort) [16]. Finally, patients in the control group were asked to methodically continue their daily routines during the total study period of one year.

### 2.4. Outcomes

Primary outcomes of the study included functional capacity and HRQoL. Functional capacity was evaluated through measurements of strength, muscle power (using a dynamometer), balance, and body positioning. HRQoL assessment was performed using the 36-Item Short Form Health Survey (SF-36), which includes bodily pain, general health, vitality, social function, emotional pain and mental health, living conditions, everyday chores, family and social relationships. All assessments were conducted only at baseline and the end of the 1-year study, and not at any intermediate stage of it. The same researcher assessed all participants at baseline and the end of the study and was blinded to patient group allocation. The assessment of the variables was conducted using the following outcomes:

1. Strength of the lower and upper extremities

An isometric dynamometer (White Plains, New York, NY, USA) was used for assessing the strength of the lower extremities, with participants positioned in a semi-squat stance and instructed to pull maximally on the handle. This procedure has been previously described by Teloudi et al. (2024), and the test has demonstrated high test–retest reliability and validity for clinical, basic, and applied research [17,18]. For the upper extremities, hand grip strength was measured, using a hand dynamometer HGS (Jamar^®^ Plus Digital Hand Dynamometer, Sammons Preston, Chicago, IL, USA). Three measurements were recorded in kg for each body site (hands and legs), and the mean value was used for each patient at each time point.

2. Flexibility of the torso

The sit-and-reach test [19] was used to assess hamstring flexibility (biceps and dorsal muscles). The tool was applied after a warm-up consisting of mild flexibility exercises, with the patients seated on the floor, shoeless, with their legs extended in front of their bodies. The patients had to reach their feet with their hands, having their hands positioned the one on top of the other and their legs in full extension. Measurements were taken during exhalation, with their hands reaching their feet, for at least 2 s. Each measurement was recorded in cm and the best measurement of the three taken in total was used for the analyses. Positive measurements indicate that the hands surpass the feet, while negative measurements denote the inability to reach their feet.

3. 30 s Sit-to-Stand (STS) Test

The 30 s sit-to-stand test is a valid tool for assessing dynamic balance and functional mobility in older adults [20]. It can also be used to evaluate muscle power of the lower extremities and general physical health [21]. During the test, patients performed the sit-to-stand-to-sit maneuver with their feet only—without use of their arms—as many times as possible in 30 s. All patients used a normal-height chair for the procedure.

4. Six-Minute Walk Test (6MWT)

The Six-Minute Walk Test (6MWT) consists of a simple, practical test used for assessing exercise functional ability and endurance in adults [22]. Its primary measurement is a 6-min walking distance (in m). During the test, patients were encouraged to walk as far as possible for 6 min in a predefined 30 m long hallway.

5. Berg balance scale (BBS)

The BBS assesses balance and risk for falls [23], particularly in older adults or people with balance issues due to neurological disease. It comprises 14 distinct tests and is performed using a timer, two standard-height chairs (one with armrests, the other without), a step/footstool and a ruler. Each test gives different scores depending on the ability of the individual to complete it. Greater scores denote better balance. The test has been previously validated and used in the Greek population [24,25].

6. Short Form Quality of Life (SF-36)

The SF-36 [26] assesses different dimensions of the health profile of each participant, by evaluating 8 domains including (1) limitations in physical activities because of health problems; (2) limitations in social activities because of physical or emotional problems; (3) limitations in usual role activities because of physical health problems; (4) bodily pain; (5) general mental health (psychological distress and well-being); (6) limitations in usual role activities because of emotional problems; (7) vitality (energy and fatigue); and (8) general health perceptions. Each domain is scored separately. Higher scores in the physical function domain indicate better capacity to conduct everyday activities easily. Lower scores in the vitality domain denote easy fatigue and low energy levels. As the Greek version of the SF-36 does not provide specific weighting coefficients for the calculation of composite scores and given that the structure of the Greek data aligns well with the American factor model, the U.S.-based scoring coefficients were used to derive the Physical and Mental Component Summary scores (PCS and MCS). This approach is consistent with methodologies adopted in other comparable studies [27]. The SF-36 questionnaire has been validated and used in the past in the Greek population, while its supplement was done by the 48 participants in the present study, who were able to understand and write, according to the questionnaire instructions and under the guidance and supervision of the researcher, where necessary.

### 2.5. Statistical Analysis

The internal consistency of the SF-36 was assessed using the Cronbach α. Additionally, answers with a negative answer were reversed and the final scoring was adjusted from 0 to 100, according to the questionnaire’s guidelines. The sample size was calculated with G^∗^Power version 3.1.9.7 (a tool to compute statistical power analyses for many different *t* tests, F tests, χ^2^ tests, z tests and some exact tests). Qualitative variables are presented as absolute and relative values and quantitative variables are presented as means and standard deviations (SD). Normality was assessed using the Kolmogorov—Smirnov and Shapiro—Wilk tests, for the total sample and each arm separately. Given that the normality assumption was not satisfied and considering the relatively small sample size, non-parametric tests were applied. Differences between groups between initial and final measurements were evaluated using the Mann–Whitney test, whereas within groups, the Wilcoxon test was used. Spearman’s rho correlation was conducted to explore relationships between the functional capacity components and the SF-36’s PCS and MCS scores. Finally, all analyses were performed using IBM SPSS Statistics (version 27). The level of significance was set at *p* < 0.05.

## 3. Results

**Figure 1:**
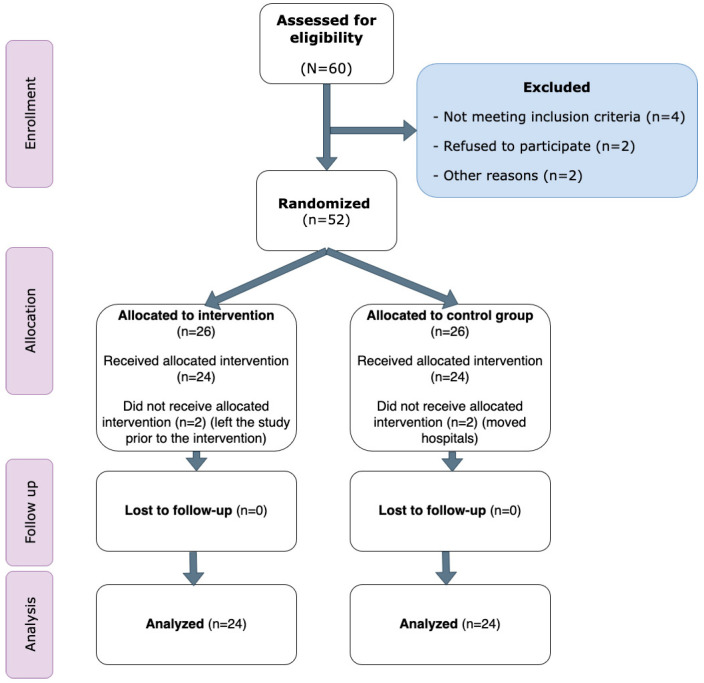
CONSORT diagram of the study.

In total, 60 patients with psychotic disorders were assessed for eligibility. Of these, four did not meet the inclusion criteria, two declined participation, and two were excluded for other reasons. Consequently, 52 patients were randomly assigned to the two study groups. After the 12-month period, 24 participants in each group remained and were included in the final analysis. No adverse events or dropouts were reported. The CONSORT diagram of the study is detailed in Figure 1 and participant characteristics are presented in Table 1. No differences were recorded between groups at baseline.

Table 2 shows the differences between groups at the end of the intervention. At the end of the 1-year study, participants in the intervention arm demonstrated increased lower and upper extremity muscle strength (*p* < 0.01), improved balance (*p* < 0.001), sit–reach and sit–stand results (*p* < 0.001), as well as increased distance covered at 6MWT (*p* < 0.001). Regarding HRQoL, all parameters of the SF-36 were improved, including general and mental health (*p* < 0.05), physical (*p* < 0.001) and social function (*p* < 0.05), role physical (*p* < 0.01) and emotional (*p* < 0.01), vitality and bodily pain (*p* < 0.001 and *p* < 0.05, respectively).

Regarding the internal consistency of the SF-36, the Cronbach’s α values were 0.8 for physical role, 0.801 for emotional role, 0.834 for vitality, 0.816 for mental health, 0.820 for social functioning, and 0.722 for bodily pain. These results indicated a good internal consistency.

To examine the associations between the components of functional capacity and the SF-36’s PSC and MCS scores, in the intervention group after the 12-month period, Spearman’s rho correlation was conducted. On the contrary, the MCS did not demonstrate statistically significant correlations with any of the functional measures, suggesting that the mental dimensions of quality of life may be affected by different factors, other than physical capacity. Detailed correlation coefficients are provided in Table 3.

## 4. Discussion

The present RCT showed that the implementation of a yearly MCET consisting of moderate-intensity aerobic exercise, Pilates exercises, and strengthening, balance, corrective and flexibility exercises performed three times per week is efficient for improving muscle strength and flexibility, balance, functional ability and HRQoL among inpatients with psychotic disorders.

The findings of the present study clearly demonstrate that a structured 12-month intervention program led to significant improvements in multiple physical fitness parameters among individuals with mental illness in the intervention group, compared to the control group. These results are consistent with international guidelines, which recognize physical activity as a key factor in promoting both physical and mental health in populations with psychiatric disorders [28].

Across multiple domains of physical fitness and psychosocial health, the findings revealed statistically significant enhancements. Specifically, lower limb strength, assessed using a dynamometer, showed a mean increase of 23.36 kg (95% Confidence Interval (CI): 15.03 to 31.7), reflecting substantial muscular improvement in the lower extremities. Such gains are particularly important for activities like walking, stair navigation, and reducing the risk of falls, especially among older adults or individuals with limited mobility. Additionally, handgrip strength increased by 5.58 kg (95% CI: 4.53 to 6.63), serving as a robust indicator of overall muscular health. Flexibility, assessed through the Sit and Reach test, demonstrated a mean improvement of 4.04 cm (95% CI: 3.04 to 5.04), indicating a notable enhancement in mobility. This gain supports greater functional independence and contributes to a reduced risk of musculoskeletal injuries. Functional endurance, measured via Sit-to-Stand repetitions, improved by 1.79 repetitions (95% CI: 1.34 to 2.24), reflecting increased muscular strength and stamina in the lower limbs—an essential factor for performing everyday tasks. Balance, evaluated using the Berg Balance Scale (BBS), showed a substantial rise of 7.38 points (95% CI: 5.61 to 9.14), highlighting a significant improvement in postural stability and a corresponding reduction in fall risk. Finally, endurance and cardiorespiratory fitness, measured by the 6-Minute Walk Test (6MWT), increased by 73.15 m (95% CI: 41.06 to 105.23), indicating better physical conditioning and an enhanced capacity for sustained physical activity.

Firth et al. [29], in the context of the Lancet Psychiatry Commission, emphasize the benefits of AE and resistance training for enhancing both physical well-being and mental functioning in individuals with psychotic disorders. In the same spirit, a recent multicenter randomized controlled trial by Maurus et al. [30] investigated the effect of AE compared to a program that included flexibility, strength, and balance exercises in individuals with schizophrenia. The results showed significant improvements in muscle strength and balance in both groups, which enhances the reliability and generalizability of our findings. This confirms that structured physical activity programs, regardless of their exact structure, can have a positive impact on the health of individuals with severe mental disorders.

The SF-36 questionnaire provided a comprehensive view of participants’ perceived health status, revealing positive changes across all HRQoL indicators. General health improved by 13.54 points (95% CI: 8.24 to 18.84), suggesting an enhanced overall health perception. Physical functioning increased by 24.27 points (95% CI: 18.07 to 30.47), indicating greater ease in performing daily activities. Improvements in role limitations due to physical health (+38.54 points, 95% CI: 21.78 to 55.3) and emotional problems (+23.61 points, 95% CI: 5.29 to 41.93) reflect better ability to manage responsibilities and increased psychological resilience. Vitality rose by 23.54 points (95% CI: 17.96 to 29.13), pointing to higher energy levels and reduced fatigue, while mental health improved by 10.17 points (95% CI: 3.37 to 16.96), signifying enhanced emotional well-being. Gains in social functioning (+9.9 points, 95% CI: 5.23 to 14.56) suggest improved interpersonal interaction, and a reduction in pain intensity or frequency was observed with an 18.33-point increase (95% CI: 12.63 to 24.04).

Previous research has also revealed that monitored, low-to-moderate-intensity ET consists of effective adjuvant treatment for patients with psychotic disorders, improving positive, negative and cognitive symptoms, as well as functioning, while reducing psychopathology and depression [12,15,31]. Regarding HRQoL, a plethora of studies [11,32], have revealed improved HRQoL with participation in ET activities among patients with schizophrenia or psychotic disorders. A recent overview of systematic reviews suggested that AE may be more effective than yoga, for reducing negative symptoms and improving HRQoL, although yoga was also found to be more effective when compared to usual treatment (no exercise) [11]. In Greece, most psychiatric hospitals do not offer the opportunity for participation in sports and exercise to inpatients. The present RCT showed that participation in ET should be incorporated in the regimes offered by Greek psychiatric hospitals, to reduce the negative symptom of weight gain induced by anti-psychotic medications and improve HRQoL and functioning of patients.

At the brain level, participation in exercise induces neurogenesis in the hippocampus while improving the synaptic plasticity [33] and increasing the concentration of various growth factors-including the brain-derived neurotrophic factor (BDNF) [34]—participating in optimal brain function maintenance [35]. Aerobic exercise tends to induce positive effects on cognitive function by improving attention and executive function, increasing the volume of brain gray matter, and improving neural neuroplasticity and connectivity [36]. In patients with schizophrenia or psychosis, lower peripheral BDNF levels are related to poorer neurocognitive functioning [37] and reduced hippocampal volume [38]. Thus, the increase in BDNF concentrations during exercise confers several improvements for patients with mental health illnesses, including improvements in the cognitive, but also the emotional and social domains [37].

The most common problem with exercise interventions is the high drop-out rate reported [15]. According to Ryu [39], it is often difficult for such patients to establish a habit for exercise in the long-term. This, however, does not constitute a limitation in the present research, possibly because the participants were inpatients at the clinic and perceived the ET program as an opportunity to engage in a different activity, occupy their time constructively and participate in a group-based intervention. In addition, the selected exercise intensity was moderate, which facilitated adherence to the program, even among older patients or those with lower fitness levels. The acceptance rate of the program was 88%. Patients in the intervention group performed 92% of the scheduled sessions. After randomization, there were no drop-outs during the 1 year and no exercise-related adverse events.

The present study was a longitudinal one; thus, improvements were inevitably anticipated in both muscle strength and flexibility. Furthermore, the present intervention lasted longer than most interventions on similar populations, and the improvements in HRQoL were also awaited. What this study showed was the use of Pilates where research is still limited. According to a meta-analysis, incorporation of Pilates exercises in patients with mental health disorders reduces depression, anxiety and the feeling of fatigue, while improving energy [40]. According to Rißmayer [12], exercise of moderate intensity, like Pilates is more efficient for improving negative symptoms, while the incorporation of mindfulness has additional a positive effect on symptoms.

Overall, the data indicate notable improvements in both the physical and mental dimensions of quality of life following the intervention. Specifically, the structured program had a significant positive effect on physical health, as reflected in the increased scores of the Physical Component Summary (PCS). In contrast, the control group showed a marked decline in physical health, potentially due to the absence of regular physical activity or other supportive measures. Regarding the Mental Component Summary (MCS), the intervention group experienced a slight improvement, while the control group demonstrated a minor decline. However, these changes were not statistically significant. This suggests that mental health may be less directly influenced by physical interventions alone or that more substantial psychological benefits may require a longer intervention period or a program specifically designed to target mental well-being.

Furthermore, the analysis revealed significant positive associations between PCS and all indicators of functional capacity, including lower-limb strength, handgrip strength, flexibility, functional mobility, balance and aerobic capacity. The strongest associations were identified between PCS and both BBS and lower-limb strength, highlighting the importance of these parameters in perceived physical health.

Several limitations of the present study merit consideration. Firstly, the effects of the exercise program on specific cardiovascular or respiratory indicators were not evaluated. Furthermore, the limited sample size in relation to the specific demographic characteristics, are also a limiting factor in the specificity of the results in terms of gender, age and body mass index. Moreover, the interpretation of outcomes concerning “quality of life”, “environment” and “social relationships” of the WHOQOL-BREF–26 scale, as well as the “social functioning” of the SF-36 scale, should be approached with circumspection, as these results may not be generalizable to the wider population of individuals with psychotic disorders. In addition, limitations related to the measurement methods and adherence may be acknowledged. Performance in functional tests could have been influenced by participants’ motivation and cooperation. Although adherence to the program was high, compliance was evaluated by the number of recorded sessions. Finally, there are sample limitations. The study population consisted of long-term hospitalized patients with psychotic disorders, characterised by prolonged institutionalization, sedentary behaviour and long periods of receiving antipsychotic medication. which may not reflect the broader population of people with psychotic disorders.

## 5. Conclusions

The results of the present study indicated that long-term MCET intervention consisting of moderate-intensity aerobic exercise, Pilates exercises, and strengthening, balance, corrective and flexibility exercises performed three times per week is feasible, safe and efficient in improving muscle strength and flexibility, balance, physical functioning and HRQoL among inpatients with psychotic disorders. These findings suggest that it could be considered a promising complementary approach to pharmacological treatment for psychotic disorders.

## Figures and Tables

**Table 1:** Characteristics of the participants.

Characteristics	Intervention Group (n = 24)	Control Group (n = 24)
Men/Women (n, %)	21 (87.5%)/3 (12.5%)	20 (83.3%)/4 (16.7%)
Age (years)	46.6 ± 13.2	47.7 ± 8.9
Psychotic disorder (n, %)		
Psychosis	8 (33.3%)	7 (29.2%)
Schizophrenia	4 (16.7%)	5 (20.8%)
schizoaffective disorder	4 (16.7%)	5 (20.8%)
delusional disorder	3 (12.5%)	2 (8.3%)
schizotypal personality disorder	2 (8.3%)	1 (4.2%)
schizophreniform disorder	2 (8.3%)	2 (8.3%)
brief psychotic disorder	1 (4.2%)	2 (8.3%)
Body mass index (BMI) (kg/m^2^)	25.8 ± 3.9	26.3 ± 3.5

**Table 2:** Differences in the outcomes between intervention and control arms at the end of the intervention (12 months).

	Intervention Group (n = 24)				Control Group (n = 24)			
Outcomes	Baseline	After 12-Months	Δ (95% CI)	Effect Size	Baseline	After 12-Months	Δ (95% CI)	Effect Size
Lower extremities strength (kg)	64.9 ± 27.2	88.2 ± 38.7 **	23.4 ± 19.7 *** (−31.7, −15)	0.61	55.0 ± 23.3	54.8 ± 23.6	−0.1 ± 7.56 (−8.2, 4.4)	0.00
Hand grip strength (kg)	23.7 ± 7.7	29.3 ± 8.7 **	−5.6 ± 2.5 *** (−6.6, 4.5)	0.61	20.9 ± 9.8	21.1 ± 9.0	0.3 ± 1.67 (−3.1, 3.3)	0.06
Sit-and-Reach (cm)	−8.4 ± 6.8	−4.4 ± 5.6 ***	−4.0 ± 2.4 *** (−5, −3)	0.61	−9.1 ± 7.9	−9.1 ± 8.1	−0.04 ± 3.0 (−1, 0.5)	0.04
Sit-to-Stand (repetitions)	9.9 ± 1.9	11.7 ± 1.4 ***	−1.8 ± 1.1 *** (−2.2, −1.3)	0.60	9.7 ± 1.2	9.8 ± 1.2	0.08 ± 0.3 (−1.2, 1.3)	0.20
BBS	40.9 ± 5.5	48.3 ± 4.3 ***	7.4 ± 4.2 *** (−9.1, −5.6)	0.60	39.7 ± 7.2	40.8 ± 6.0	1.1 ± 2.6 (−4.3, 9.1)	0.32
6MWT (m)	279.5 ± 77.4	352.6 ± 77.8 ***	−73.2 ± 76.0 *** (−105.2, 41.1)	0.61	244.5 ± 65.3	242.1 ± 69.1	−2.4 ± 15.8 (−0.2, 0)	0.07
SF-36 General health	55.4 ± 14.4	69.0 ± 19.9 *	13.5 ± 12.6 *** (−18.8, −8.2)	0.52	54.8 ± 19.3	56.7 ± 17.2	1.9 ± 14.9 (−4.4, 8.2)	0.05
SF-36 Physical function	60.3 ± 20.7	84.6 ± 11.2 ***	24.3 ± 14.7 *** (−30.5, −18.1)	0.61	61.4 ± 22.4	62.0 ± 16.9	0.6 ± 17.4 (−6.7, 7.9)	0.00
SF-36 Role physical	46.9 ± 37.8	85.4 ± 25.5 **	38.5 ± 39.7 *** (−55.3, −21.8)	0.51	47.9 ± 42.3	49.0 ± 41.4	1.0 ± 34.2 (−13.4, 15.4)	0.00
SF-36 Role emotional	40.3 ± 42.8	63.9 ± 42.8 **	23.6 ± 43.4 ** (−41.9, −5.3)	0.34	43.1 ± 42.3	45.8 ± 45.9	1.4 ± 34.7 (−13.3, 16.1)	0.04
SF-36 Vitality	43.5 ± 14.3	67.1 ± 22.0 ***	23.5 ± 13.2 *** (−29.1, −18.0)	0.61	44.4 ± 15.6	45.0 ± 16.3	0.6 ± 11.7 (−4.3, 5.5)	0.04
SF-36 Mental health	49.5 ± 9.1	59.7 ± 19.7 *	10.2 ± 16.1 * (−17.0, −3.4)	0.38	47.5 ± 14.5	49.0 ± 13.8	0.7 ± 12.8 (−4.7, 6.1)	0.13
SF-36 Social functioning	62.5 ± 16.5	72.4 ± 16.9 *	9.9 ± 11.0 ** (−14.6, −5.2)	0.47	59.9 ± 25.8	58.9 ± 24.9	−1.0 ± 19.5 (−9.2, 7.2)	0.08
SF-36 Bodily pain	60.8 ± 15.1	79.2 ± 17.6 *	18.3 ± 13.5 *** (−24, −12.6)	0.59	62.9 ± 22.7	63.8 ± 21.5	+0.8 ± 17.8 (−6.7, 8.3)	0.03
PCS	49.7 ± 9.0	54.6 ± 7.0 **	4.9 ± 7.2 *** (−8, −1.8)	0.44	50.3 ± 9.7	45.4 ± 7.8	−4.9 ± 6.4 (−2.2, 0)	0.44
MCS	50.0 ± 5.8	51.2 ± 7.2	1.2 ± 6.9 (−4.1, −1.7)	0.15	50 ± 10.1	48.8 ± 8	−1.2 ± 9.0 (−2.6, 5)	0.06

6MWT: 6-Minute Walk Test; BBS: Berg Balance Scale; SF-36: 36-Item Short Form Health Survey; PCS: Physical Component Summary Score; MSC: Mental Component Summary score. * Significantly different compared to the intervention arm at the same time point. *** *p* < 0.001; ** *p* < 0.01; * *p* < 0.05.

**Table 3:** Correlation (Spearman’s rho) between Outcomes and Physical and Mental Component Summary.

Outcomes	PCS	MCS
Lower extremities strength (kg)	0.556 **	−0.091
Hand grip strength (kg)	0.473 *	0.034
Sit-and-Reach (cm)	0.497 *	0.100
Sit-to-Stand (repetitions)	0.417 *	0.090
BBS	0.592 **	0.068
6MWT (m)	0.475 *	0.061

6MWT: 6-Minute Walk Test; BBS: Berg Balance Scale; PCS: Physical Component Summary Score; MSC: Mental Component Summary score. ** Correlation is significant at the 0.01 level (2-tailed). * Correlation is significant at the 0.05 level (2-tailed).

## Data Availability

The original contributions presented in this study are included in the article. Further inquiries can be directed to the corresponding author.

## Abbreviations

ET	Exercise training program
BBS	Berg balance scale
HRQoL	Health-related quality of life
MCS	Mental component summary score
MECT	Multi-component exercise training
PCS	Physical component summary score
SF-36	36-item short form health survey
STS	Sit-to-stand test
6MWT	Six-minute walk test
